# The making of a maggot brain

**DOI:** 10.7554/eLife.86696

**Published:** 2023-03-03

**Authors:** Andreas S Thum, Bertram Gerber

**Affiliations:** 1 Institute of Biology, University of Leipzig Leipzig Germany; 2 Department Genetics of Learning and Memory, Leibniz Institute for Neurobiology Magdeburg Germany

**Keywords:** metamorphosis, mushroom body, trans-differentiation, brain, neurons, interneurons, *D. melanogaster*

## Abstract

The way neurons in the brain rewire in larvae as they turn to adult fruit flies sheds light on how complete metamorphosis was ‘invented’ over the course of evolution.

**Related research article** Truman JW, Price J, Miyares RL, Lee T. 2023. Metamorphosis of memory circuits in *Drosophila* reveals a strategy for evolving a larval brain. *eLife* **12**:e80594. doi: 10.7554/eLife.80594.

Want a new life? And a new body? What sounds like a Faustian bargain is business as usual for animals with a complete metamorphosis, such as butterflies, bees or fruit flies. Once hatched from an egg, these insects spend their ‘first life’ as soft-bodied crawling larvae, dedicated to feeding and growing, and their ‘second life’ as hard-shelled adult insects with genitalia and wings, dedicated to reproduction.

The evolutionary origins of such a remarkable, complete metamorphosis, and how it happens over the course of an animal’s development, has fascinated humankind for more than 2,000 years (Truman, 2019; Reynolds, 2019). Evolutionarily ancestral insects undergo no such metamorphosis (Truman and Riddiford, 2019; Belles, 2019). Juvenile grasshoppers, for example, do not have a larval phase and instead emerge as miniature adults that lack wings and working reproductive organs. They then molt multiple times before developing into a full-size, sexually mature adult (Figure 1A). More modern insects, however, do not transition from a larva to an adult in such a continuous manner: when their larval life ends, they establish a pupal case to allow the larval body to dissolve and the adult body to take shape. Complete metamorphosis thus required evolving a detour from the direct, evolutionarily ancestral trajectory to first form the larva, and then returning back to the ancestral pathway to develop the adult body (Figure 1B).

**Figure 1.**
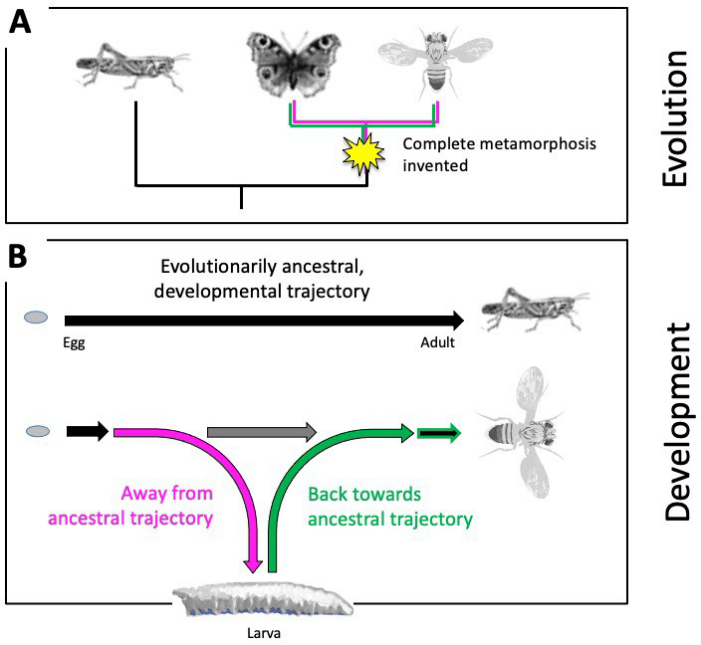
Evolutionary origins of metamorphosis. (**A**) Complete metamorphosis – where an animal goes through a larval and pupal life stage before developing into an adult – appeared relatively late in the insect evolutionary family tree during the advent of winged insects from the *Pterygota* family. It is the defining feature of a group of insects known as the *Holometabola*, which includes butterflies and flies (shown on the right). Hemimetabolous insects, such as grasshoppers (shown on the left), on the other hand, emerge as nymphs that look like adults (except for their lack of wings and genitalia), and gradually develop into mature adults after a series of molts. (**B**) Top: Development in evolutionarily ancestral insects, such as grasshoppers, follows a straight trajectory (black line). From the egg hatches a miniature and sexually immature version of the adult that eventually develops via multiple growth molts (not shown). Bottom: In insects with complete metamorphosis, development involves a detour away from the ancestral trajectory (magenta) to establish a larva. Development resumes back towards the evolutionarily ancestral trajectory (green) via a pupal stage (not shown), giving rise to the adult fly. The horizontal grey arrow indicates the so-called imaginal discs – cells that are stored and arrested early during development – to be available later for establishing the adult fly.

This, however, poses a problem for the development of the brain. Ancestral insects with a direct development trajectory essentially possess a miniature adult brain as soon as they hatch. But making a larval stage requires adjusting the brain and the connections within it to this new, larval way of life. Now, in eLife, James Truman, Jacquelyn Price, Rosa Miyares, and Tzumin Lee from the Howard Hughes Medical Institute report new insights into the process of metamorphosis by studying the brains of fruit flies (Truman et al., 2023).

To find out more about how brain circuits change during metamorphosis, the researchers focused on a brain structure important for learning and memory in insects, called the mushroom body, and the interneurons located within this region. Their experiments revealed a stunning diversity of fates as these interneurons go through this turbulent process. Some cells die and are replaced, while others survive metamorphosis and become integrated into the adult mushroom body in much the same way as in the larva. Other neurons survive and remain in the mushroom body but are integrated in a completely different way, and some survive but have a completely different role in a different brain region.

One hurdle larvae face is that when development detours from the ancestral trajectory, few neurons are mature enough to assume a functional role. Indeed, the number of functional neurons in the larva is only about a tenth of the adult number. To make a larval brain that is nevertheless functionally complete, cellular redundancy is sacrificed wherever possible, i.e., specific tasks that require multiple neurons working in parallel in adults, are served by only one neuron in the larva. The problem, however, is that some neurons are functionally necessary but not yet mature, whereas others are mature but not needed.

The solutions can be spectacularly pragmatic. For example, a neuron known as MBON-g1 is stopped in its tracks towards its adult, ancestral fate and is instead recruited to the larval mushroom body, where it is involved in associative learning. When development resumes the ancestral trajectory (i.e., as the pupa morphs into an adult), tMBON-g1 retreats from the mushroom body and takes on its original mission in another, now rapidly developing brain structure for navigation, called the central complex.

As a result of the diverse fates neurons can take on during metamorphosis, the anatomical relationships between the modulatory input and output neurons of the mushroom body are profoundly altered during the pupal stage. These relationships are important for associative learning, and it therefore seems as if associative memories established during larval life would be ‘deliberately’ wiped out – or would at least get massively reconfigured – as development resumes (Aso et al., 2014a; Aso et al., 2014b, Saumweber et al., 2018).

Although the connections between the specific neurons change, important general features of the mushroom body do not. For example, learning about rewards and punishments takes place in segregated regional lobes of the mushroom body in both the larva and adult fly.

Recent studies have further shown that a gene called *chinmo* – together with two other genes – organizes the developmental rollercoaster that allows to make a larva, which then transitions, via the pupal stage, to an adult fly (Truman and Riddiford, 2022; Williams and Kafatos, 1971). Together, these studies provide valuable insight into the evolutionary making of two lives out of one. However, exactly how this gene trio ensures that the neurons in the brain are properly remodeled remains to be determined (Meltzer and Schuldiner, 2020).

It would also be interesting to see if it is possible to pinpoint the specific mutations in these genes that eventually led to the evolution of complete metamorphosis, and how these genetic mechanisms compare to other animals going through a similar transition, such as some amphibians. Would this uncover similar principles for animals cycling through sessile and mobile stages, such as cnidarians, or through different hosts, such as parasites? Clearly, studies investigating how developmental processes change over the course of evolution (like the paper by Truman et al.) have a bright future.
